# Simultaneous Structural Identification of Natural Products in Fractions of Crude Extract of the Rare Endangered Plant *Anoectochilus roxburghii* Using ^1^H NMR/RRLC-MS Parallel Dynamic Spectroscopy

**DOI:** 10.3390/ijms12042556

**Published:** 2011-04-15

**Authors:** Xiao-Xue Wang, Jiu-Ming He, Chun-Lan Wang, Rui-Ping Zhang, Wen-Yi He, Shun-Xing Guo, Rui-Xiang Sun, Zeper Abliz

**Affiliations:** 1 Key Laboratory of Bioactive Substances and Resource Utilization of Chinese Herbal Medicine, Ministry of Education, Institute of Materia Medica, Chinese Academy of Medical Sciences and Peking Union Medical College, Beijing 100050, China; E-Mails: waitgirl@imm.ac.cn (X.-X.W.); hejiuming@imm.ac.cn (J.-M.H.); rpzhang@imm.ac.cn (R.-P.Z.); hewy@imm.ac.cn (W.-Y.H.); 2 Institute of Medicinal Plant Development, Chinese Academy of Medical Sciences and Peking Union Medical College, Beijing 100094, China; E-Mails: wangchunlan2006@163.com (C.-L.W.); sxguo2006@yahoo.com.cn (S.-X.G.); 3 Institute of Computing Technology, Chinese Academy of Sciences, Beijing 100080, China; E-Mail: rxsun@ict.ac.cn

**Keywords:** ^1^H NMR/RRLC-MS parallel dynamic spectroscopy, flavonoids, organic acids, crude extract, *Anoectochilus roburghii*

## Abstract

Nuclear magnetic resonance/liquid chromatography-mass spectroscopy parallel dynamic spectroscopy (NMR/LC-MS PDS) is a method aimed at the simultaneous structural identification of natural products in complex mixtures. In this study, the method is illustrated with respect to ^1^H NMR and rapid resolution liquid chromatography-mass spectroscopy (RRLC-MS) data, acquired from the crude extract of *Anoectochilus roxburghii*, which was separated into a series of fractions with the concentration of constituent dynamic variation using reversed-phase preparative chromatography. Through fraction ranges and intensity changing profiles in ^1^H NMR/RRLC–MS PDS spectrum, ^1^H NMR and the extracted ion chromatogram (XIC) signals deriving from the same individual constituent, were correlated due to the signal amplitude co-variation resulting from the concentration variation of constituents in a series of incompletely separated fractions. 1H NMR/RRLC-MS PDS was then successfully used to identify three types of natural products, including eight flavonoids, four organic acids and p-hydroxybenzaldehyde, five of which have not previously been reported in *Anoectochilus roxburghii*. In addition, two groups of co-eluted compounds were successfully identified. The results prove that this approach should be of benefit in the unequivocal structural determination of a variety of classes of compounds from extremely complex mixtures, such as herbs and biological samples, which will lead to improved efficiency in the identification of new potential lead compounds.

## 1. Introduction

The discovery and identification of new chemical entities in complex mixtures means a ubiquitous challenge in drug discovery and development regimes. To accomplish the time-consuming task of screening pharmaceutical libraries, often consisting of (multi)millions of molecules, a large variety of methodologies is currently available [1]. During the last few years, we have reported a series of methods for the rapid structural identification of compounds in crude extracts from herbal medicines and Traditional Chinese Medicine by MS/MS and liquid chromatography-MS/MS (LC-MS/MS) [2–10]. However, sometimes nuclear magnetic resonance (NMR) data (mostly ^1^H NMR) was needed to provide complementary information for the purpose of validating the chemical structures adequately, especially in complex mixture analysis. In fact, parallel use of NMR and MS methods as structural tools have efficiently provided complementary data in structural elucidation studies for natural product research, drug metabolite analysis, and other complex mixture analysis problems [11–13]. Recently, directly coupled LC-NMR-MS has been used in pharmaceutical laboratories worldwide to avoid traditional isolation of analytes [14–16]. Successful studies have also been conducted using HPLC-NMR-MS, allowing a superior level of peak discrimination and structure elucidation [17–20]. However, some technical drawbacks still exist in direct hyphenated methods involving the use of NMR [21,22], such as high cost, a narrow range of deuterated solvents as LC eluents, lower detection sensitivity, and the compatibility of the chromatographic peak volume with that of the NMR flow cell.

Alternatively, the combination of chemometric and mathematical methods relying on inherent multivariate profiling capabilities have been successfully used to recover latent active compound information, such as potential biomarkers in metabonomics. Recently, a number of statistical techniques have aided in peak resolution and identification, such as statistical total correlation spectroscopy (STOCSY) [23], statistical heterospectroscopy (SHY) [24] and NMR/LC–MS parallel dynamic spectroscopy (NMR/LC–MS PDS). In particular, NMR/LC–MS PDS, based on the off-line analysis of a series of incompletely separated chromatographic fractions with different concentration changing profiles of the constituents, can provide the intrinsic correlation between retention time (Rt), mass/charge (*m/z*) and chemical shift (δ) data of the same individual constituent in the LC fractions through the co-analysis of visualized MS and NMR data with signal amplitude covariation in the NMR/LC–MS PDS spectra. As a consequence, the complementary spectral information is obtained from mixture spectra for unambiguous structural identification of individual constituents in crude extracts [25]. Using NMR/LC-MS PDS, the complementary strengths of the two methods can be combined, and the covisualization of NMR and MS data can yield not only the simplification of the separation analysis procedure for complex mixtures, but also simultaneous and unambiguous structural information than can be either used alone or applied pairwise between individual samples.

The genera Anoectochilus and Goodyera (Orchidaceae) are perennial herbs which comprise more than 35 species and are widespread in the tropical regions, from India through the Himalayas and Southeast Asia to Hawaii [26]. Of those species, *Anoectochilus roburghii*, an indigenous and valuable Chinese folk medicine, has been used as a popular nutraceutical herbal tea in China and other Asian countries. This herbal plant is also called ‘king medicine’ because of its diverse pharmacological effects. The whole dried plants have been widely used to treat diabetes [27], cancers [28], underdeveloped children [29], liver diseases [30], cardiovascular diseases [31], nephritis and venomous snake bite [32], *etc.*, in China, further proving that natural products should be considered important resources for future medicines which Koop [33] advocated in his article in *Science*. Reports are available on the constituents of the herb, which include flavonoids, organic acids and aliphatic compounds, and both flavonoids and glucosides were found to be the predominant components [34–36]. Because of a low budding and growth rate in natural surroundings, predatory mass collection, and damages to the ecological environment, the natural resources of *Anoectochilus roburghii* are becoming exhausted. Thus, artificial breeding ones of this species by tissue culture techniques are gradually commercialized as substitutes used for the same purpose in the recent marketplace [37]. Therefore, the rapid and simultaneous structural identification of natural products in the wild plant has become very important to assess the quality of the cultivars. It is known that precious and endangered materials such as *Anoectochilus roxburghii* and *Taxus madia* are very difficult to obtain or can be obtained in small quantities. Therefore, considering that the major advantage of NMR/LC-MS PDS over routine separation and structural elucidation methodologies is that fewer samples as well as analysis time are needed, we used this method as a structural identification tool to identify natural products in *Anoectochilus roburghii.*

Previous work has shown that NMR/LC–MS PDS successfully identified identical types of natural products, such as 12 flavonol glycosides in an active herbal extract from flowers of *Gossypium herbaceam* L [25] and 7 phenylethanoid glycosides in the crude extract of *Forsythia suspensa* [38]. In this work, we placed particular emphasis on the simultaneous structure of variety classes of compounds, and presented the results obtained from a crude extract of the rare endangered plant *Anoectochilus roburghii.* Here, a reversed-phase preparative column chromatography was employed to simplify the separation procedure and acquire a series of fractions with different concentrations from complex mixtures, while flash column chromatography, which is unpopular and has moderate separation, was used in previous work. Moreover, RRLC and microcoil probe were first employed in this method to improve signal resolution and sensitivity. Finally, the study involved a ^1^H NMR/RRLC–MS PDS analysis of ^1^H NMR spectra and available negative RRLC-MS spectra of the fractions, together with fragmentation behavior analysis of MS/MS spectra acquired in the same instrumental run to develop a relatively rapid, precise and accurate method for the structural identification of different types of compounds in complex samples.

## 2. Results and Discussion

### 2.1. RRLC-MS Analysis of the Crude Extract

**Figure 1.**
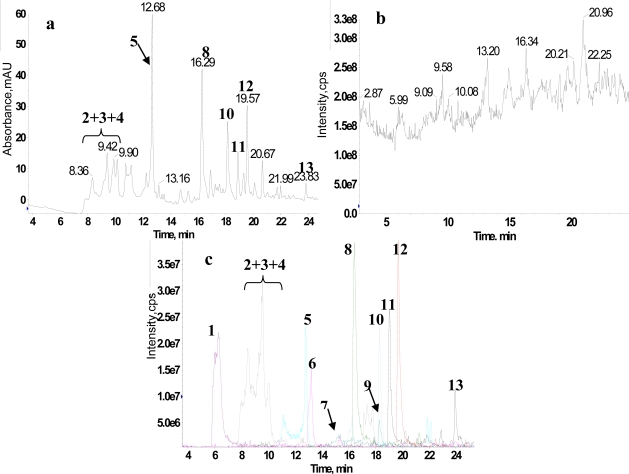
**(a)** HPLC-UV chromatogram of the crude extract at λ 345 nm; **(b)** Total ion chromatogram of RRLC-MS in negative ion mode; **(c)** Extracted ion chromatogram of the crude extract.

We chose RRLC as a separation tool for the analysis of the crude extract to achieve an increase in separation efficiency, shorter run times than HPLC, and better peak resolution [39]. Moreover, it was reported that there were a large number of flavonoid glycosides in *Anoectochilus roxburghii*, therefore a wavelength of UV detector at 345 nm for the UV detection was chosen as the detection wavelength. Figure 1(a) shows the RRLC-UV chromatogram spectrum for the crude extract. We found that the retention times of the main compounds in the crude extract were between 6 and 24 min. Considering that some compounds without chromophore groups have low UV absorption intensity, the total ion chromatography (TIC) spectrum of the crude extract is shown in Figure 1(b). Based on the results of the ^1^H NMR/RRLC-MS PDS spectrum which is discussed in detail below, the extracted ion chromatogram (XIC) spectra of 13 compounds are shown in Figure 1(c), and the 13 compounds including the main compounds shown in Figure 1(a) were numbered in order by their retention times in RRLC-MS. It was apparent that the relative contents of these compounds varied widely and some compounds, including a group of isomers, were eluted together with almost non-separation, which indicated that the crude extract was very complex.

**Figure 2.**
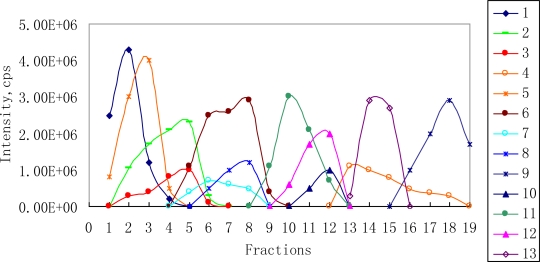
Scatter diagram of the composition profiles of the 13 constituents in the series of fractions from the ethanol extract reconstructed from RRLC-MS analysis.

Figure 2 displays the composition profiles of the 13 constituents, which was reconstructed by plotting the XIC areas of all the constituents in the series of fractions. This clearly showed that the crude extract was incompletely separated, and the 13 constituents eluted into nearly different fractions and processed different concentration changing profiles. Most importantly, compound 4 and 9 were eluted into different fractions with their co-eluted compounds, respectively, and were considerably hard to separate in the analysis of the crude extract even when an excellent separation tool (RRLC) was used (Figure 1(c)). These results suggested that preparative column chromatography could be applied in the separation of extremely complex herbal extracts into an incompletely separated series of fractions.

### 2.2. ^1^H NMR/RRLC-MS PDS Spectrum

**Figure 3.**
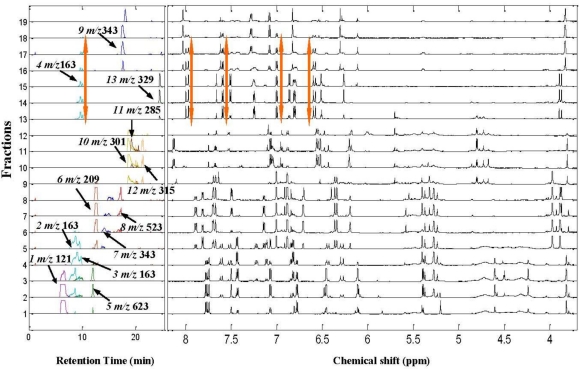
^1^H NMR/RRLC-MS PDS spectrum of the crude extract of *Anoectochilus roxburghii*.

**Figure 4.**
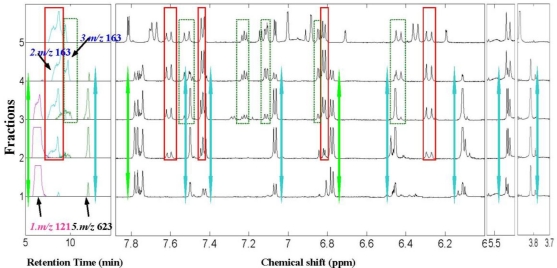
Enlarged ^1^H NMR/RRLC-MS PDS spectrum from fraction 1 to 5.

A series of fractions were taken for ^1^H NMR and RRLC-MS analysis, and after data processing the signal amplitude co-variation between the ^1^H NMR and XICs signals were visualized together to produce the ^1^H NMR/RRLC-MS PDS spectrum of the ethanol extract of *Anoectochilus roxburghii* (shown in Figure 3). For our applications, the fraction axis (vertical axis) resembled the retention time axis in the chromatogram, and each line represented the XIC and ^1^H NMR spectra of each fraction. It can be seen that the XICs signals of the 13 constituents including two groups of isomers were lined out on the ^1^H NMR/RRLC-MS PDS spectrum with suitable separation and eluting in different fractions. Figure 3 shows that, constituent 9 and 10, which were strongly overlapped in the XIC spectrum of the ethanol extract were eluted into different fractions and could be distinguished clearly by the ^1^H NMR/RRLC-MS PDS spectrum with dark blue and yellow profiles respectively. In addition, one of the three isomers with [M–H]^−^ ion at *m/z* 163, constituent 4, could also be clearly distinguished from the other two isomers (constituents 2 and 3) owing to its distribution in different fraction ranges, which indicated that this approach could play a prominent role in the chemical structural identification of co-eluted constituents. Based on this visualization tool, the intrinsic correlation between ^1^H NMR and RRLC-MS data of the same constituent could be discovered easily, such as constituent 4 with orange arrows highlighting the mass/charge (*m/z)* and chemical shifts (δ) data. In order to illustrate the significant role of the ^1^H NMR/RRLC-MS PDS spectrum in the structural identification of individual constituents in a complex mixture, we magnified the ^1^H NMR/RRLC-MS PDS spectrum of the crude extract from fraction 1 to 5 (Figure 4), in which ^1^H NMR signals of four constituents were highlighted in the same colored square or arrow with corresponding XICs, respectively, and two co-eluted isomers with critically overlapped peaks appeared.

**Figure 5.**
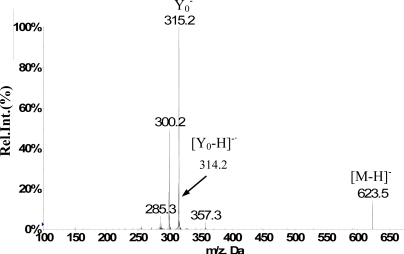
RRLC-MS/MS spectrum of [M-H]^−^ at *m/z* 623 corresponds to constituent 5.

It can be clearly observed in Figure 4 that constituent 5 with [M-H]^−^ at *m/z* 623 (highlighted with blue arrow) was eluted at a Rt of 12.1 min with signal amplitude dynamic variation from fractions 1 to 4. Although constituent 1 with [M–H]^−^ at *m/z* 121 shared the same fraction range with constituent 5, the two compounds presented different dynamic concentration changing profiles as shown in Figure 2. Thus, the ^1^H NMR signals of the correct compound could be picked out and assigned. Based on the co-variation among the fraction ranges and signal intensity changing profiles, six columns of ^1^H NMR signals (highlighted with blue arrows) varied in almost the same fraction range with the XIC at *m/z* 623 and were correlated and assigned to constituent 5. Then, through using the correlated XIC and ^1^H NMR signals as index and comparing their fraction ranges, a doublet of doublet at δ 7.40 ppm was recognized as a seriously overlapped signal with that of another constituent from fractions 2 to 4 along fraction axis. However, in fraction 1, it was a pure signal of constituent 5 with coupling constants of 2.0 and 8.0 Hz, displaying correlation with two recovered doublets at δ 7.50 and 7.06 ppm with coupling constants of 2.0 and 8.0 Hz respectively, which indicated a typical ABX coupling system of 3’,4’-disubstituted ring B of a flavonoid skeleton. Two doublets at δ 6.42 and 6.11 ppm with the same coupling constant of 2.0 Hz which were partly overlapped in fractions 1 and 2 by other signals were attributed to two protons at the meta position of disubstituted ring A. A singlet at δ 3.83 ppm was recognized as a signal of a methoxyl group. Therefore, the skeleton of constituent 5 was presumed to be isorhamnetin. The supplementary RRLC-MS/MS spectrum corresponding to *m/z* 623 shown in Figure 5(a) was helpful, giving significant fragment peaks at *m/z* 315, 314, 300 and 285. Previous work has shown the collision-induced disassociation of flavonoid-3-*O*-[6″-rhamnosyl(1→6)]-glucoside, displaying a neutral loss of 308 Da (146 + 162 Da) from [M-H]^−^ ion and high abundance Y_0_^−^ ion with lower [Y_0_-H]^−^ ion observed at the same time [40]. Constituent 5 matched the relevant *m/z* value with *m/z* 315 and 314. For that reason, we can speculate that constituent 5 was substituted by a rutinoside group at position 3. Therefore, the structure isorhamnetin3-*O*-[6″-rhamnosyl(1→6)]-glucopyranoside which has been identified in *Anoectochilus roxburghii* [41] was assigned to compound 5. In addition, this flavonoid was a good example of how the ^1^H NMR/RRLC-MS PDS spectrum with supplementary RRLC-MS/MS spectrum was used to deconvolve overlapping NMR peaks and to support the identification of the substituent positions of glycosyl groups on flavonoids.

Not only did the ^1^H NMR/RRLC-MS PDS spectrum with incompleted separation strategy have a significant role in the assignment of overlapping signals, it was also important in the structural identification of co-eluted isomers, when the chromatographic separation conditions were carefully optimized in the hyphenated NMR technique. For example, three peaks were observed in the “blue” XIC of [M-H]^−^ ion at *m/z* 163, and named constituent 2, 3 and 4, respectively, in Figure 3. Of these, the first two constituents were incompletely separated with almost the same retention time and XICs fraction range from fraction 2 to 5 (shown in Figure 2), which resulted in great difficulty in distinguishing and identifying their chemical structures. However, detailed inspection of Figure 4 revealed that, benefited from the lower sensitivity of ^1^H NMR to MS, most of the ^1^H NMR signals of constituent 3 with lower concentration appeared from fraction 3 to 5, different from that of constituent 2. Therefore, the ^1^H NMR signals could easily be attributed to the correct compound. Interestingly, constituent 4, nearly co-eluted with the other isomers in RRLC-MS analysis of the crude extract as shown in Figure 1(c), was eluted into different fractions from constituents 2 and 3 by preparative column chromatography, which also facilitated the elucidation of the three compounds. In Figure 4, four sets of ^1^H NMR signals (highlighted with red squares), that is δ 7.44 (2H, *J* = 8.6 Hz), 6.80 (2H, *J* = 8.6 Hz), 7.59 (*J* = 16.0 Hz), and 6.27 ppm (*J* = 16.0 Hz), varied in the same fraction ranges with the XIC at *m/z* 163 were correlated and assigned to constituent 2. Although the doublet at δ 7.44 ppm was seriously overlapped with a doublet of doublet from constituent 3 in fractions 2 and 3 along fraction axis, it was a pure signal of constituent 2 in fractions 4 and 5. Then, the first two doublets were attributed to two pairs of protons with the same chemical shift respectively at adjacent position of a benzene ring while the latter two doublets were attributed to two protons across the double band from each other. RRLC-MS/MS data corresponding to *m/z* 163 (constituent 2) listed in Table 1 indicated the presence of a carboxyl group by the characteristic product ions at *m/z* 119, the putative decarboxylated species, a very reasonable neutral loss (44 Da) from the parent ion at *m/z* 163. Comparing the ^1^H NMR signals with those of trans-4-hydroxycinnamic acid previously reported as a *Anoectochilus roxburghii* constituent in a literature [34], we found a high degree of consensus. Therefore, constituent 2 was identified as trans-4-hydroxycinnamic acid. Again, the ^1^H NMR/RRLC-MS PDS method deconvolved the overlapping NMR peaks, in this case revealing an organic acid. As for constituent 3, three columns of ^1^H NMR signals (highlighted with green squares) at δ 7.23, 7.10, and 6.85 ppm, covering almost the same fraction range with the XIC peak corresponding to constituent 3, were picked out first. Subsequently, two doublets at δ 7.52 and 6.43 ppm were recognized and assigned to constituent 3 by detailed inspection of the ^1^H NMR/RRLC-MS PDS spectrum. Moreover, the relevant RRLC-MS/MS signals corresponding to *m/z* 163 (constituent 3) listed in Table 1 was highly similar to that of compound 2, giving significant product ions at *m/z* 119 and 93. The above data indicated that constituent 3 and 2 shared the same groups just with different substituent sites. Thus, despite deficiency in the deconvolution of a seriously overlapping NMR peak from an aromatic proton, useful correlated data were enough to identify constituent 3 as trans-3-hydroxycinnamic acid, which was first discovered in *Anoectochilus roxburghii.* Signal assignments of constituent 3 are presented in Table 1. In Figure 3, the fraction range and intensity changing profile of each XIC and ^1^H NMR signal from constituent 4 could be observed clearly and highlighted with orange arrows.

**Figure 6.**
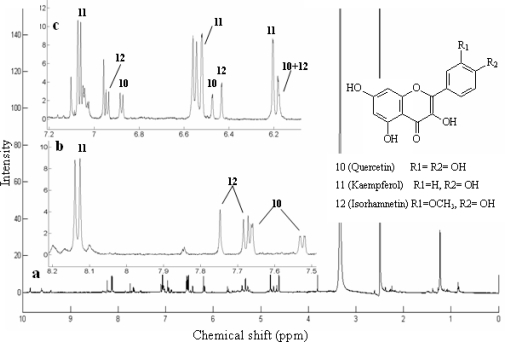
^1^H NMR spectrum of fraction 11 from the crude extract and the ^1^H NMR signals were correlated and assigned using NMR/LC-MS PDS. **(a)** Full spectrum; **(b)** same spectrum between 7.5 and 8.2 ppm; **(c)** same spectrum between 6.1 and 7.2 ppm.

In Figure 3, three peaks in the XIC of *m/z* 301, 285 and 315 covered almost the same fractions 9–12, which resulted from the co-elution of constituent 10, 11 and 12. The assignment of ^1^H NMR peaks was difficult due to the overlap of parts of the signals. However, using NMR/LC-MS PDS spectrum, the problem can be approached as follows. Four ^1^H NMR peaks at the high-frequency region were clearly presented at δ 8.15 (2H, *J* = 8.8 Hz), 7.07 (2H, *J* = 8.8 Hz), 6.53 (*J* = 2.0 Hz) and 6.27 ppm (*J* = 2.0 Hz), respectively, as observed in the relevant ^1^H NMR spectra of fraction 11 shown in Figure 6. Subsequently, the signal amplitude dynamic co-variation between the four peaks and the XIC of *m/z* 301, 285, 315 was co-analyzed. We found that the intensity variation of the above ^1^H NMR signals from fractions 9 to 11 and that of the XIC peaks at *m/z* 285 showed the same tendency. Consequently, the columns of the six protons were assigned to constituent 11. In the same way, the other mixed and overlapped protons signals from fractions 9 to 12 were correlated and deconvolved simultaneously, and were assigned to constituents 10 and 12, respectively. Figure 6 shows the complete NMR signal assignment to constituents 10, 11 and 12 in fraction 11. Finally, three flavone aglycones, quercetin, isorhamnetin and kaempferol, with similar structure were identified unambiguously based on the ^1^H NMR/RRLC-MS PDS spectrum by co-analyzing their ^1^H NMR peaks, the XIC signals. To our surprise, kaempferol, a most familiar constituent, has not been reported in *Anoectochilus roxburghii.* In addition, the assignment of –OH signals of quercetin were listed in Table 1 following the results of reported literatures [42,43].

Taking advantage of the ^1^H NMR/RRLC-MS PDS spectrum, the complementary RRLC-MS and ^1^H NMR data for the 13 constituents in the crude extract were correlated and recovered successfully for unambiguous structure identification, and further reinforced with corresponding supplementary information from RRLC-MS/MS spectra. Eight flavonoids (constituents 5, and 7–13) and four organic acids (constituents 2–4, and 6), and *p*-hydroxybenzaldehyde (constituent 1) were identified. The recovered RRLC-MS data, ^1^H NMR data, product ions, retention times and molecular formula are listed in Table 1, and ^1^H NMR spectra with primary signal assignments are presented in the supplementary information.

## 3. Experimental Section

### 3.1. Reagents

*Anoectochilus roxburghii* was collected from Fujian, China, and was identified by Prof. GUO shun-xing, Chinese Academy of Medical Sciences and Peking Union Medical College. The dried and powdered whole herbs were then extracted with 95% v/v ethanol in water and concentrated *in vacuo* to yield a crude extract applied by Prof. GUO shun-xing. HPLC-grade acetonitrile and formic acid were obtained from Merck (Darmstadt, Germany). Dimethyl sulfoxide-D6 (DMSO) containing 0.03% (v/v) tetramethylsilane (TMS) was obtained from Cambridge Isotope Laboratories Inc.

### 3.2. Fractions Preparation

The crude extract (0.55 g) was dissolved in 5 mL of a mixed solution containing acetonitrile and water (4:1 by volume). Preparative column chromatography separations were performed on a 15 cm ×19 mm reversed-phase C18 (Waters SunFire™) 5-μm column at room temperature, using an elution of water (eluent A) and acetonitrile (eluent B) at a flow rate of 10 mL/min for 90 min. The composition was started at 5% B, and then ramped linearly to 100% B at 90 min. The crude extract was separated into a series of fractions collected at a fixed time interval of 1 min. 0.2mL of each fraction was taken for RRLC-MS analysis and the remaining fractions were dried by rotary evaporation.

### 3.3. Instrumentation and Conditions

#### 3.3.1. RRLC-MS and RRLC-MS/MS Analysis

RRLC-MS analysis was performed using an Agilent 1200 RRLC series HPLC system (Agilent Technologies, Waldbronn, Germany) coupled to the QTRAP MS spectrometer (QTRAP 2000, Applied Biosystems/MDS SCIEX) tandem mass spectrometer equipped with a Turbo Ion spray ion source (Concord, ON, Canada) which was controlled by Analyst 1.5. Fractions of 5 μL were injected onto a 2.1 mm × 100 mm reversed-phase Zorbax SB-C18 1.8-μm RRLC column maintained in an oven at 50 °C. The column was eluted under gradient conditions at a flow rate of 200 μL/min for a duration of 31 min, then changed to 250 μL/min for the last 9min; mobile phase component A consisted of 0.1% aqueous formic acid, and component B consisted of acetonitrile. Gradient conditions were as follows: 0–10 min linear gradient 5–30% B, 10–20 min linear gradient 30–50% B, 20–21 min 50–60% B, 21–30 min 60–95% B, 30–31 min 95–100% B, 31–40 min 100–100% B, then returned to 5% B and re-equilibrated over the final 10 min prior to injection of the next sample. UV spectra were recorded from 190 to 400 nm and the detection wavelength was set at 345 nm. The mass spectrometer was operated in negative ion mode with an ionspray voltage of −4.5 kV, declustering potential of −70 V, curtain gas of 25 (arbitrary units), nebulizer gas (gas1) flow of 70 (arbitrary units), and heater gas (gas2) of 60 (arbitrary units). The source temperature was set at 350 °C. Spectra were collected in the enhanced full mass scan mode from *m/z* 50–1000. RRLC-MS/MS analysis was performed using the same LC conditions as above. For MS/MS, the collision gas was N_2_ and set at high, collision energies were −35eV in the enhanced product ion (EPI) scan. In the linear ion trap (LIT) mode, the scan speed was 1000 Da/s and the LIT fill time was set at 80 ms, and only quadrupole (Q0) trapping was activated while EPI data were acquired, the r.f./DC analyzing quadrupole (Q1) was set at unit mass resolution. Spectra were collected in the enhanced product ion (EPI) scan mode from *m/z* 50–800.

#### 3.3.2. NMR Samples and Analysis

19 fractions from the incompleted separation fractions were selected for ^1^H NMR analysis according to the LC-MS analysis results. Before NMR analysis, the dried residuals were then transferred to 3-mm NMR tubes with acetonitrile/water 4:1 (v/v), respectively, dried completely in a nitrogen stream and redissolved in 0.2 mL DMSO for NMR analysis.

All NMR spectra were acquired at 599.7 MHz on a Varian NMR System-600 NMR spectrometer using a 3-mm SW probe controlled by Varian Vnmr 6.0 C software software. 3-mm NMR tubes (NO. S-3–600–7) were purchased from NORELL Inc., to obtain high-resolution spectra. For each sample, 32 free induction decays (FIDs) were collected into 34,374 data points at a spectral width of 8400 Hz, with an acquisition time of 13 min per sample and a 1 s relaxation delay, and all scans were acquired at 298 K.

### 3.4. Data Processing

Total ion chromatogram (TIC) data from RRLC–MS were converted into MATLAB format file (ms.mat) in Analyst 1.5, and the RRLC-UV data were converted into text format file. Then the potential [M−H]^−^ ions were extracted to produce extracted ion chromatogram (XIC) data by in-house routines written in MATLAB 7.0.1 (Mathworks, Natick, MA, USA).

^1^H NMR spectra from Varian format data files were phased, baseline corrected, smoothed and referenced to TMS (δ 0.0) using MestReC 4.9.9.6 after an exponential line-broadening factor of 0.3 Hz was applied to the FIDs prior to Fourier transformation, and then exported as ASCII format file (nmr.txt). The ASCII format files were read into MATLAB for threshold setting. Sections of the 1H NMR spectra containing the aromatic and aliphatic signals were considered.

## 4. Conclusions

Following its original application in an active extract obtained from *Gossypium herbaceam L.*, the results presented here demonstrate the general usefulness of NMR/LC-MS PDS for extracting structural information on components present in a crude extract based on ^1^H NMR and RRLC-MS, using a hybrid mass spectrometer (QLIT) with a suitable IDA protocol. Applied to the rare endangered plant *Anoectochilus roxburghii*, this approach with some technological modifications enables the synthesis of ^1^H NMR spectra, XIC signals and MS/MS data and successfully permits the identification of different types of compounds in the extract. Furthermore, the results of this method can be expected to significantly aid in comparing the constituents of *Anoectochilus roxburghii* with those which were cultivated by biological techniques, which will be addressed in future publications. Overall, ^1^H NMR/RRLC-MS PDS combined with an incompleted separation strategy has an important future role in expediting the structural identification of constituents in crude extracts, and indeed for the research of covering an even greater variety of different target molecules in complex samples.

## Figures and Tables

**Table 1.** RRLC-MS and ^1^H NMR data of 13 constituents correlated and recovered using ^1^H NMR/RRLC-MS PDS method.

**Compound**	**Rt (min)**	**[M-H]^−^ (*m/z*)**	**^1^H NMR data (δ, ppm; *J*, Hz)**	**LC-MS/MS data (*m/z*, relative abundances, ℅)**
*p*-hydroxybenzaldehyde (1)	6.8	121	H_–CHO_ 9.87s, H-2,6 7.76d (2H, 8.5Hz), H-3,5 6.78d (2H, 8.5Hz)	121(100), 92(97), 65(10)
trans-4-hydroxycinnamic acid (2)	8.5	163	H_–OH_ 9.78brs, H-2,6 7.44d (2H, 8.6 Hz), H-3,5 6.80d (2H, 8.6 Hz), H-7 7.59 d (16.0 Hz), H-8 6.27d (16.0 Hz)	163(10), 119(100), 93(20)
trans-3-hydroxycinnamic acid (3)*	9.2	163	H-2 7.03a, H-4 6.85dd (2.0, 8.5Hz), H-5 7.23t (8.5,8.5Hz), H-6 7.10a(8.5,2.0Hz), H-7 6.43d(15.9Hz), H-8 7.52d (15.9Hz)	163(10), 119(100), 93(20)
trans-2-hydroxycinnamic acid (4)*	9.7	163	H-3 7.00d (8.1Hz), H-4 6.88 m, H-5 7.24 m, H-6 7.59d (7.7Hz), H-7 7.99 d (16Hz), H-8 6.59d (16Hz)	163(10), 119(100), 93(30)
Isorhamnetin-3-*O*-[6″-rhamnosyl(1→6)] glucopyranoside(5)	12.1	623	H-2’ 7.50 d (2.0Hz), H-5’ 7.06 d (8.5Hz), H-6’ 7.41dd (2.0,8.5Hz), H-6 6.11 d(2.0Hz), H-8 6.45d (2.0Hz), H-1″ 5.39a, H_–OCH3_ 3.83s (3H)	623(25), 357(10), 315(100), 300(50), 285(10)
5-hydroxyferulic acid (6)*	13.0	209	H-2 7.00d(1.5Hz), H-6 6.88d(1.5Hz), H-7 7.69d (15.5Hz), H-8 6.33d(15.5Hz), H_–OCH3_ 3.97s (3H)	209(70), 165(40), 141(100)
3,5-dihydroxy-3’,4’,7-trimethoflavone (7)	15.0	343	H-6’7.85dd (2.0,8.5Hz), H-2’7.77 d (2.0Hz), H_–OCH3_ 3.84 s (9H), H-5’7.14 d (8.5Hz), H-8 6.71 d (2.0Hz), H-6 6.19 d (2.0Hz)	313(90), 285(15), 254(50), 242(100), 198(30),151(10)
5,6,3’,4’-tetrahydroxy-7,5’-trimethoflavonol-3’-*O*-glucoside (8)*	16.2	523	H-2’ 7.50d(1.5Hz), H-6’ 7.48d (1.5Hz), H-8 6.91s, H_–OCH3_ 3.89s (3H), 3.87s (3H)	523(70), 361(100), 329(30), 315(40), 299(20), 179(10)
5,4’-dihydroxy-6,7,3’-trimethoflavone (9)	18.4	343	H-2’8.03d (1.5H_Z_), H-6’7.29dd (1.5,8.0Hz), H 5’7.08d (8.0Hz), H-8 6.82s, H-3 6.30s H_–OCH3_ 3.84s (9H)	343(25), 328(49), 313(100), 298(10), 242(37), 214(8)
Quercetin (10)	18.4	301	H-2’7.65d (2.0Hz), H-5’6.88d (8.5Hz), H-8 6.47d (2.0Hz), H-6’7.53dd (2.0,8.5Hz), H-6 6.18 d (2.0Hz), H-3’-OH 9.35brs, H-3-OH 9.40 brs, H-4’-OH 9.65 brs	301(84), 273(7), 179(40), 151(100), 121(26), 107(43)
Kaempferol (11)*	18.9	285	H-2 ‘,6’ 8.15 d (2H, 8.8 Hz), H-3’,5’7.07 d (2H, 8.8 Hz), H-8 6.53 d (2.0 Hz), H-6 6.27d(2.0 Hz)	285(100), 229(6), 211(7),185(8)
Isorhamnetin (12)	19.6	315	H-2’ 7.74s, H-6’ 7.68d (8.5Hz), H-5’ 6.93d (8.5Hz), H-6 6.18d (2.0Hz),H-8 6.43 d (2.0Hz), H_–OCH3_3.81s (3H)	315(90), 300(100), 271(12), 255(10), 227(15), 164(20), 151(35)
Quercetin 3,4’-dimethyl ether (13)	24.6	329	H-2’7.66d (2.0Hz), H-6’7.51dd (2.0, 8.5Hz), H-5’6.87d (8.5Hz), H-8 6.51d (1.8Hz), H-6 6.26d (1.8Hz), H_–OCH3_ 3.89s (3H), 3.87s (3H)	329(70), 314(64), 299(45), 285(58), 271(95), 243(100), 199(22)

a, multiplicity of signals; s, singlet; d, doublet; m, multiplet; brs, broad singlet.

* first discovered in *Anoectochilus roxburghii*.
